# Constitutive Modelling Analysis and Hot Deformation Process of AISI 8822H Steel

**DOI:** 10.3390/ma17235713

**Published:** 2024-11-22

**Authors:** Khaled Elanany, Wojciech Borek, Saad Ebied

**Affiliations:** 1 Silesian University of Technology, 2A Akademicka Str., 44-100 Gliwice, Poland; ke900594@student.polsl.pl; 2 Department of Engineering Materials and Biomaterials, Silesian University of Technology, 18A Konarskiego Str., 44-100 Gliwice, Poland; 3 Department of Production Engineering and Mechanical Design, Faculty of Engineering, Tanta University, Tanta 31527, Egypt; saad_ebied@f-eng.tanta.edu.eg

**Keywords:** AISI 8822H steel, hot deformation, strain rate, temperature, flow stress, Gleeble 3800, constitutive models

## Abstract

This study used the Gleeble 3800 thermomechanical simulator to examine the hot deformation characteristics of AISI 8822H steel. The main goal was to understand the alloy’s behaviour under various thermomechanical settings, emphasising temperature ranges between 1173 K and 1323 K and strain rates from 0.01 s^−1^ to 10 s^−1^. This study aimed to enhance the alloy’s manufacturing process by offering a thorough understanding of the material’s response to these conditions. Four various constitutive models—Arrhenius-type, Johnson–Cook, modified Johnson–Cook, and Trimble—were used in a comprehensive technique to forecast flow stress values in order to meet the study’s goals. The accuracy of each model in forecasting the behaviour of the material under the given circumstances was assessed. A thorough comparison investigation revealed that the Trimble model was the most accurate model allowing prediction of material behaviour, with the maximum correlation factor (R = 0.99) and at least average absolute relative error (1.7%). On the other hand, the Johnson–Cook model had the least correlation factor (R = 0.92) and the maximum average absolute relative error (32.2%), indicating that it was the least accurate because it could not account for all softening effects.

## 1. Introduction

Steel alloys are fundamental to technological and industrial advancements due to their versatility, strength, and customizable properties [1]. Composed primarily of iron and carbon, steel alloys can include additional elements to enhance specific characteristics [2]. For example, carbon increases hardness and strength, manganese improves wear resistance, nickel improves impact and corrosion resistance, and chromium boosts corrosion resistance [3]. To obtain the required qualities, iron ore must be melted, impurities must be eliminated, and alloying materials have to be added [4]. Tool steel, stainless steel, and carbon steel are the three types of steel that are suitable for different uses. Carbon steel is prevalent in structural and automotive uses; stainless steel is ideal for medical equipment and kitchenware due to its corrosion resistance; and tool steel’s remarkable strength and heat endurance make it suitable for cutting tools. The adaptability and robust properties of steel alloys make them indispensable in industries like manufacturing, aerospace, construction, and medical equipment [5,6,7,8,9].

AISI 8822H is a nickel–chromium–molybdenum steel classified under H-steel grades, known for its high strength and toughness [10]. Its specific composition enhances its properties beyond standard carbon steels, making it ideal for mechanical applications [11]. It is commonly used for manufacturing crankshafts, gears, fasteners, axles, and shafts, all requiring high strength and hardness. Owing to its endurance and resistance to wear, this alloy is also appropriate for heavy-duty machinery and tools [12]. AISI 8822H is especially valuable in applications needing superior strength-to-weight ratios and outstanding mechanical performance, such as drilling tools and equipment used in extreme environments [13]. Forging and extrusion and other hot-forming processes can be used to further shape the structure and properties of this material and thus enable its use in new areas of industry.

Numerous constitutive models have been published to illustrate the flow stress behaviours of various alloys throughout temperature and strain rate ranges. The Arrhenius-type hyperbolic sine model, introduced by Sellars and Tegart [14], along with Zener and Hollomon [15], is one of the most common and earliest constitutive models. This model determines the relationship linking stress and strain rate, making it particularly suitable for elevated temperatures [16]. One of the main limitations of the Arrhenius model is its inability to consider strain. To take into account the strain effect and evaluate the flow stress behaviour in 42CrMO steel, Lin et al. developed an adjusted Arrhenius-type model incorporating strain compensation [17].

Another common constitutive model is the Johnson–Cook model. This model determines the alloy’s flow stress behaviour during hot deformation testing using adiabatic and isothermal circumstances. Nevertheless, just one strain rate was used to study the impact of temperature [18]. To refine the initial Johnson–Cook model constants for the 2024Al-T351 alloy, Adibi-Sedeh et al. [19] carried out machining operations.

Based on actual compression data, Maheshwari et al. introduced an improved version of the Johnson–Cook model [20] to explain the flow stress characteristics of 2024Al alloy. The modified model was found to correlate better with experimental data in most scenarios compared to the original version. Maheshwari [21] developed a novel phenomenological constitutive model with a significantly higher correlation with data from experiments than the modified Johnson–Cook model previously provided. Khan and Liu [22] conducted compression tests on 2024Al-T351 alloy samples and, based on the results, proposed a novel phenomenological model describing the behaviour of flow stresses. Building on the original version of the Johnson–Cook model, LIN et al. [23] proposed an additional constitutive approach to forecast the deformation behaviour of Al-Zn-Mg-Cu, Al-Cu-Mg [24], and 7075Al [25] alloys during hot tensile tests. The authors observed higher prediction accuracy when compared to the Johnson–Cook model. To describe the flow stress behaviour of 7075Al, the researchers Trimble and O’Donnell [26] developed a newer model with a unique approach to constitutive modelling, described in detail in the research section of this article.

The main goal was to understand the alloy’s behaviour under various thermomechanical settings, emphasising temperature ranges between 1173 K and 1323 K and strain rates from 0.01 s^−1^ to 10 s^−1^. This study aimed to enhance the alloy’s manufacturing process by offering a thorough understanding of the material’s response to these conditions. Four various constitutive models—Arrhenius-type, Johnson–Cook, modified Johnson–Cook, and Trimble—were used in a comprehensive technique to forecast flow stress values in order to meet the study’s goals.

## 2. Experiments

**Figure 1:**
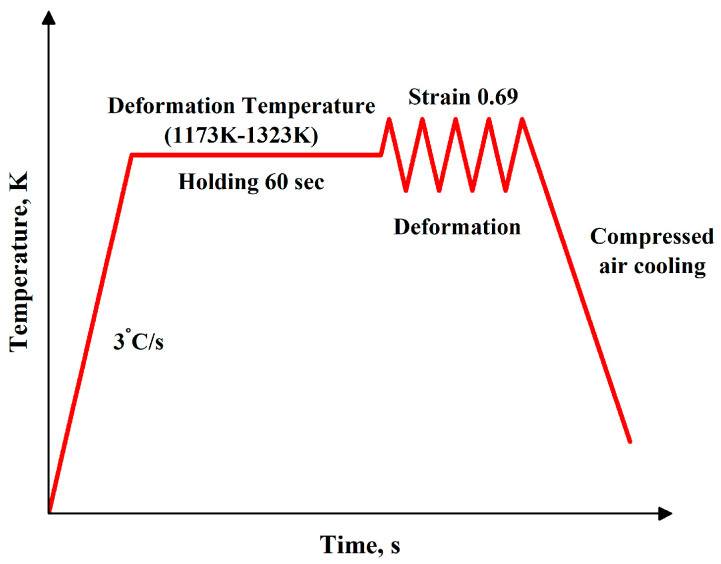
Schematic illustration representing the hot deformation process of AISI 8822H steel using the Gleeble simulator.

The test samples were manufactured in a cylindrical shape, measuring 10 mm in diameter, 12 mm in length, and 12 mm in height. Table 1 shows the weight proportion of the AISI 8822H alloy’s chemical composition [10]. The Gleeble 3800 thermomechanical simulator was used to conduct the hot uniaxial compression experiments at four distinct temperatures (1173, 1223, 1273, and 1323 K) and strain rates (0.01, 0.1, 1, and 10 s^−1^). To guarantee a consistent temperature distribution, the sample was heated to the deformation temperature with a heating rate of 3 °C/s and isothermal hold for one minute before being compressed, as illustrated in Figure 1. The Gleeble 3800 (Dynamic Systems Inc., Poughkeepsie, NY, USA) is equipped with direct resistance heating. This simulator is capable of heating samples at heating rates of over 10,000 °C/s and maintaining the temperature to within ±1 °C. The compressed air quenching procedure was used to maintain the deformed microstructure as soon as the samples were exposed to an actual true strain of 0.69. High-temperature nickel-based grease was applied to tantalum foils and on the contact surface of the sample to minimise friction between the sample tungsten carbide anvils. Type K thermocouple wires were used to monitor temperature throughout the test. For the hot compression test, ORIGIN PRO^®^ 2024 was used to create true strain–true stress curves.

## 3. Results

**Figure 2:**
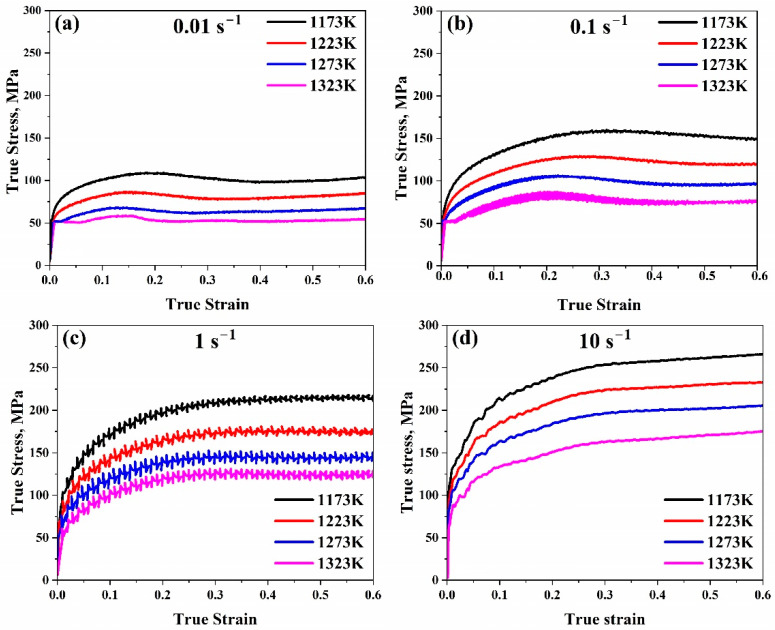
True strain–stress curves of AISI 8822H steel at strain rates (**a**) 0.01 s^−1^, (**b**) 0.1 s^−1^, (**c**) 1 s^−1^, (**d**) 10 s^−1^.

Figure 2 displays typical true strain–true stress curves of the AISI 8822H alloy at various temperatures (1173, 1223, 1273, and 1323 K) and strain rates (0.01, 0.1, 1, and 10 s^−1^) attained during the hot compression experiments. The curves in this figure were used to retrieve the flow stress values at different temperatures and strain rates within the true strain range of 0.2–0.6.

Due to work hardening, independent of the strain rate at initial strains, the flow stress increased with increasing strain under all test conditions and temperatures. The initial application of strain causes subgrain formation and an increase in dislocation density. The maximum stress and work hardening rate increased as expected with decreasing temperature and increasing strain rate.

The maximum stresses in the curves can be observed in the strain range of 0.1–0.3, with minor variations depending on strain rate and deformation temperature. The flow stress shows a characteristic maximum value of stress conservation at low strain rates of 0.01 and 0.1 s^−1^ for all temperatures. This is followed by flow softening with further straining. The flow stress curves displayed the usual behaviour of maximum stress peak and flow softening followed by a steady state, especially at lower strain rates (0.01–0.1 s^−1^) and higher temperatures (1273–1323 K).

Shared softening features at low temperatures (usually 1173 K) and low strain rates (0.01 s^−1^) could be explained by flow localisation. However, a subsequent increase in strain rate led to the flow localisation gradually disappearing as the flow stress behaviour attained stability, a feature of dynamic recovery (DRV). The flow curves should be able to show that they are approaching a steady state at 1 s^−1^. Because of dynamic recovery, the steady-state flow imitates a dynamic equilibrium between flow softening and strain hardening. However, a decrease in the flow is observed around 10 s^−1^, which may be caused by adiabatic heating. During the heated deformation phase, an adiabatic temperature rise is encouraged at a high strain rate of 10 s^−1^. At the same strain rate, as a result, the flow stress curves decrease, particularly at low temperatures.

The flow stress behaviour of the AISI 8822H alloy was studied using four constitutive models at a strain step of 0.05. The models covered a temperature range of 1173–1323 K, a strain range of (0.2–0.6), and a strain rate range of 0.01–10 s^−1^. The Arrhenius-based [14,15], original and modified Johnson–Cook [20,21], and Trimble models [26] were employed in this investigation. Following an assessment of each model’s output, a comparison of experimental and anticipated flow stress levels was formed between the four models, determining which was better suited to assess the most accurate data.

### 3.1. Arrhenius-Type Model

The general formula of this model, as shown in Equation (1) [14], can be separated into three different forms depending on the level of stress [15], as shown in Equations (2)–(4).

(1) $$\dot{\varepsilon }=AF\left(\sigma \right)exp\left(-\frac{Q_{def}}{RT}\right)$$

(2) $$\dot{\varepsilon }=A_{1}\sigma^{n^{\prime }}\exp\;\left(-\frac{Q_{def}}{RT}\right),\alpha \sigma <0.8$$

(3) $$\dot{\varepsilon }=A_{2}\exp\left(\beta \sigma \right)\exp\;\left(-\frac{Q_{def}}{RT}\right),\alpha \sigma >1.2$$

(4) $$\dot{\varepsilon }=A{\left[sinh\left(\alpha \sigma \right)\right]}^{n}\exp\;\left(-\frac{Q_{def}}{RT}\right),for\;all\;stresses$$

where σ is the flow stress (MPa); $\dot{\varepsilon }$ is the strain rate (s^−1^); $Q_{def}$ is the activation energy (J/mol); R is the universal gas constant (8.314 J/(mol·K)); T is the temperature (K); $A,\;A_{1},\;A_{2},\alpha ,\;\beta$ are material constants; and $n,\;n^{\prime }$ are strain indices.

**Figure 3:**
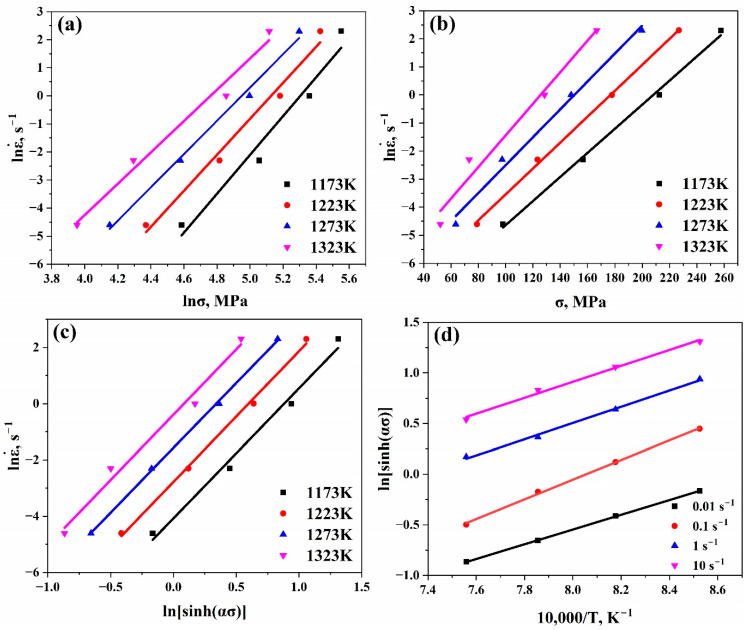
Plots of (**a**) $\left(ln\dot{\varepsilon }\;vs.\;ln\sigma \right)$, (**b**) $\left(ln\dot{\varepsilon }\;vs.\;\sigma \right)$, (**c**) $\left(ln\dot{\varepsilon }\;vs.ln\left[sinh\left(\alpha \sigma \right)\right]\right)$, (**d**) $\left(ln[sinh(\alpha \sigma )]\;vs.\;10000/T\right)$ to evaluate $n^{\prime }$, β, n, and s, respectively, at 0.4 ε.

Equations (2) and (3) were used to evaluate $n^{\prime }$ and β, respectively, by taking natural logarithm in both sides, as shown in Equations (5) and (6). $n^{\prime }$ and β could be calculated as the mean slopes’ values of the linear fits of the curves $\left(ln\dot{\varepsilon }\;vs.\;ln\sigma \right)$ and $\left(ln\dot{\varepsilon }\;vs.\;\sigma \right)$, as shown in Figure 3a and Figure 3b, respectively. Therefore, α might be estimated using Equation (7).

(5) $$ln\varepsilon ˙=lnA_{1}+n^{\prime }ln\sigma -\frac{Q_{def}}{RT}$$

(6) $$ln\varepsilon ˙=lnA_{2}+\beta \sigma -\frac{Q_{def}}{RT}$$

(7) $$\alpha =\beta /n^{\prime }$$

After that, n was estimated using Equation (8) as the mean slopes’ values of the linear fits of the curve $\left(ln\dot{\varepsilon }\;vs.\;ln\left[sinh\left(\alpha \sigma \right)\right]\right)$, as shown in Figure 3c.

(8) $$ln\varepsilon ˙=lnA+n\;ln[sinh\left(\alpha \sigma \right)]-\frac{Q_{def}}{RT}$$

In the next step, a secondary constant s was estimated using Equation (9) as the mean slopes’ values of the linear fits of the curve $\left(ln[sinh(\alpha \sigma )]\;vs.\;1/T\right)$, as shown in Figure 3d. And, therefore, given n and R, Q could be estimated using Equation (10).

(9) $$\frac{Q_{def}}{Rn}=\frac{\partial ln[sinh(\alpha \sigma )]}{\partial (1/T)}=s$$

(10) $$Q_{def}=Rns$$

**Figure 4:**
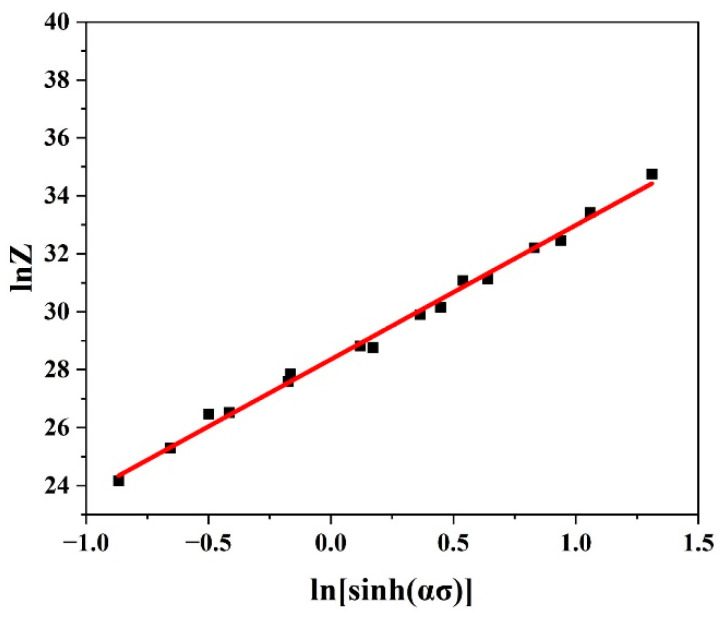
Plots of $\left(lnZ\;vs.\;ln[sinh(\alpha \sigma )]\right)$ to evaluate lnA at 0.4 ε.

Equation (11) illustrates the temperature-compensated strain rate *Z* that Zener and Hollomon established. To obtain A, the intercept of the linear fit of the curve $\left(lnZ\;vs.\;ln[sinh(\alpha \sigma )]\right)$ was found to provide lnA by taking the natural logarithm for each side, as seen in Figure 4, and consequently, A could be estimated.

(11) $$Z=\dot{\varepsilon }exp\;(\frac{Q_{def}}{RT})=A{\left[sinh\left(\alpha \sigma \right)\right]}^{n}$$

Using Figure 3 and Figure 4, the values of $n^{\prime }$, β, α, n, $Q_{def}\;(kJ/\mathrm{mol})$, and A at 0.4 ε could be evaluated as listed in Table 2.

Using Equation (11), the Arrhenius-type constitutive formula can be recast as follows:

(12) $$\sigma =\frac{1}{\alpha }ln\left\{{\left(\frac{Z}{A}\right)}^{\frac{1}{n}}+{\left[{\left(\frac{Z}{A}\right)}^{\frac{2}{n}}+1\right]}^{\frac{1}{2}}\right\}$$

Using Equation (12), the constitutive equations for all strains can be stated as follows:

(13) $$\sigma_{0.4}=\frac{1}{0.008}ln\left\{{\left(\frac{Z_{0.4}}{2*10^{12}}\right)}^{\frac{1}{4.6}}+{\left[{\left(\frac{Z_{0.2}}{2*10^{12}}\right)}^{\frac{2}{4.6}}+1\right]}^{\frac{1}{2}}\right\}$$

Repeating all the previous steps at a strain range of (0.2–0.6), five values of $n^{\prime }$, β, α, n, $Q_{def}$, and A could then be obtained. These values could be used to define all constitutive equations at the strain range. By substituting *Z* values into the constitutive equations, the predicted flow stress values at all temperature, strain, and strain rate ranges could be calculated.

**Figure 5:**
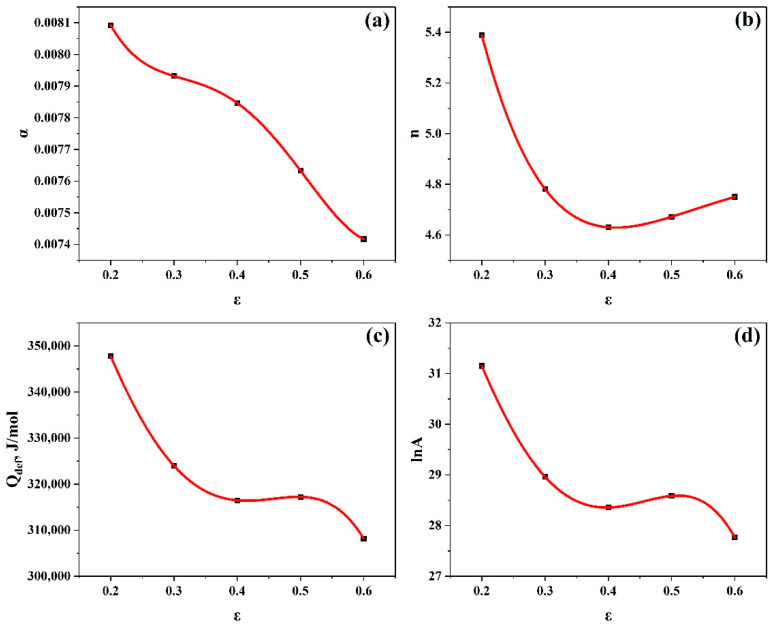
Polynomial fitting of (**a**) αvs.ε, (**b**)n vs. ε, (**c**) $Q_{def}\;vs.\;\varepsilon$, (**d**) lnA vs.ε.

A polynomial fit was produced for each constant evaluated under different strains, and it was demonstrated that the best match was achieved when the fourth order of polynomials was used, as illustrated in Figure 5.

As a result, the regression in the equations of the relevant material constants α, n, $Q_{def}$, and lnA as a function of strain was reasonably described by Equations (14)–(17), respectively.


(14) $$\alpha =0.0111-0.03348\varepsilon +0.13187\varepsilon^{2}-0.22624\varepsilon^{3}+0.13731\varepsilon^{4}$$

(15) $$n=9.55976-35.76636\varepsilon +94.07557\varepsilon^{2}-106.60521\varepsilon^{3}+44.83021\varepsilon^{4}$$

(16) $$Q_{def}=4.26*10^{5}-3.47*10^{5}\varepsilon -9.6*10^{5}\varepsilon^{2}+4.53*10^{6}\varepsilon^{3}-4.19*10^{6}\varepsilon^{4}$$

(17) $$lnA=37.54237-20.44644\varepsilon -146.89034\varepsilon^{2}+541.78232\varepsilon^{3}-475.74717\varepsilon^{4}$$

Using the correlation coefficient (*R*) and average absolute relative error (*AARE*), the predicted flow stress deviation was assessed to compare the predictability of such a constitutive model.

The correlation coefficient illustrates the significance of the linear correlation of the experimental and predicted values. It should be noted that an elevated (*R*) value does not always suggest better performance because the model tends to be biased toward higher or lower values. However, the average absolute relative error (*AARE*) is a statistical measure that may be used to objectively assess a model’s predictability because it is computed by comparing the relative deviations term by term [15,27,28].

**Figure 6:**
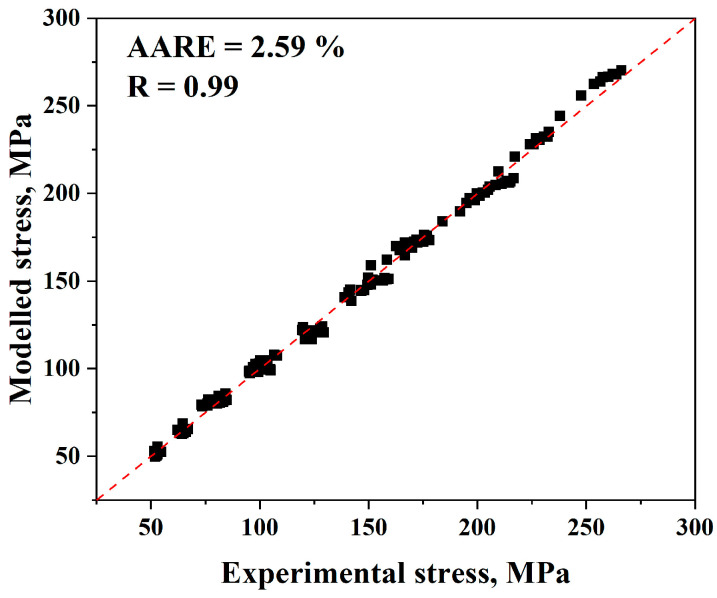
Comparison of the Arrhenius-type model’s anticipated and experimental stress data.

Equations (18) and (19) can be used to express (*AARE*) and (*R*). For the Arrhenius-type model, the values of (*AARE*) and (*R*) are 2.59% and 0.99, respectively, as shown in Figure 6.


(18) $$AARE=\frac{1}{N}\sum_{i=1}^{N}\left|\frac{E_{i}-P_{i}}{E_{i}}\right|\times 100\%$$

(19) $$R=\frac{\sum_{i=1}^{N}\left(E_{i}-\bar{E}\right)\left(P_{i}-\bar{P}\right)}{\sqrt{\sum_{i=1}^{N}{\left(E_{i}-\bar{E}\right)}^{2}\sum_{i=1}^{N}{\left(P_{i}-\bar{P}\right)}^{2}}}$$

**Figure 7:**
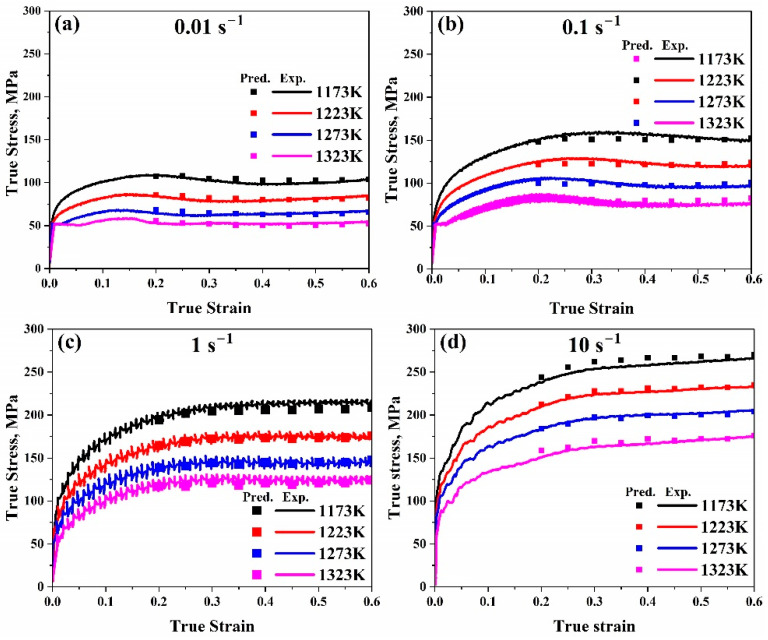
Comparison of the Arrhenius-type model’s true strain–true stress anticipated and experimental flow stress data at temperature range (1173–1323 K) at (**a**) 0.01 s^−1^, (**b**) 0.1 s^−1^, (**c**) 1 s^−1^, and (**d**) 10 s^−1^, respectively.

As illustrated in Figure 7, the strain–stress experimental curves could be compared with the predicted flow stress values after evaluation.

### 3.2. Johnson–Cook Model (J–C)

An illustration of the Johnson–Cook model is as follows [16] in Equation (20):

(20) $$\sigma =\left(A_{j}+B_{j}\varepsilon^{n_{j}}\right)\left(1+C_{j}ln{\dot{\varepsilon }}^{*}\right)\left(1-{\left({T_{j}}^{*}\right)}^{m_{j}}\right)$$

where ε is the strain; σ is the flow stress (MPa); ${\dot{\varepsilon }}^{*}$ is the dimensionless strain rate $\left({\dot{\varepsilon }}^{*}=\dot{\varepsilon }/{\dot{\varepsilon }}_{ο}\right)$; $\dot{\varepsilon }$ is the strain rate (s^−1^); ${\dot{\varepsilon }}_{ο}$ is the reference strain rate (s^−1^); ${T_{j}}^{*}$ equals to $\left(T-T_{r}\right)/\left(T_{m}-T_{r}\right)$; T is the current temperature (K); $T_{m}$ is the melting temperature (K); $T_{r}$ is the reference temperature (K); $A_{j}$ is the yield strength at the reference strain rate and temperature; and $B_{j},\;C_{j},n_{j},\;m_{j}$ are material constants.

Since the temperature range is (1173–1323 K), the reference temperature, 1173 K, was assumed to be the lowest value of this range. The reference strain rate was assumed to be 1 s^−1^. Moreover, as shown in Figure 2c, the AISI 8822H alloy yield stress at reference conditions is approximately 60 MPa. It was estimated that our alloy melts around 1743 K. Yield stress, melting temperature, and reference values are exceptionally important for solving this model’s general formula, as mentioned in Equation (20).

Equation (20) can be rewritten as follows by multiplying both sides by the natural logarithm at the reference conditions:

(21) $$\ln\;\left(\sigma -A_{j}\right)=lnB_{j}+n_{j}ln\varepsilon$$

**Figure 8:**
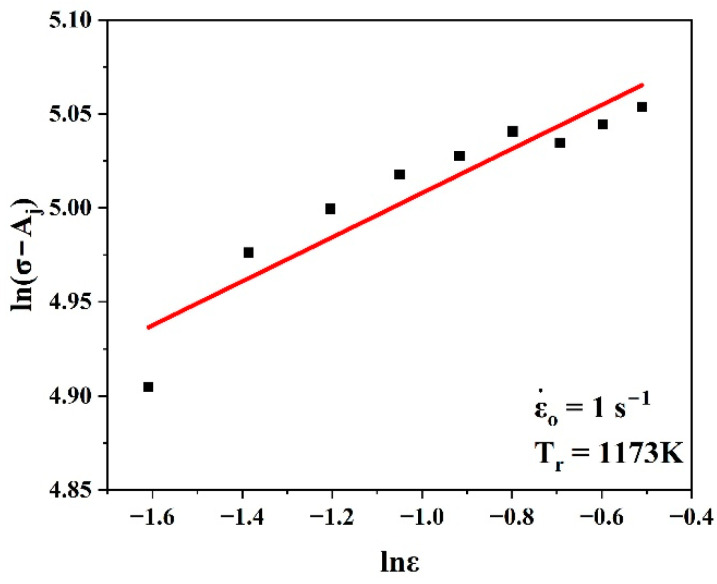
Plots of $ln(\sigma -A_{j})\;vs.\;ln\varepsilon$ to evaluate $n_{j}$ and $lnB_{j}$.

Equation (21) was used to evaluate $n_{j}$ and $lnB_{j}$ by taking the slope and intercept of the linear fit of the curve $ln(\sigma -A_{j})\;vs.\;ln\varepsilon$, as shown in Figure 8. Hence, it was simple to evaluate $B_{j}$.

Equation (20) can be modified to the following at reference temperature:

(22) $$\frac{\sigma }{\left(A_{j}+B_{j}\varepsilon^{n_{j}}\right)}-1=C_{j}ln{\dot{\varepsilon }}^{*}$$

**Figure 9:**
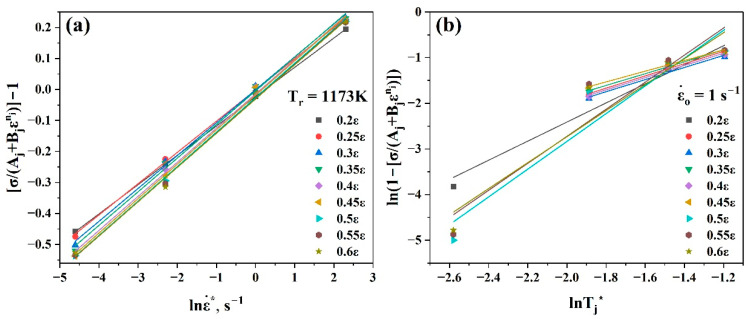
Plots of (**a**) $\frac{\sigma }{\left(A_{j}+B_{j}\varepsilon^{n_{j}}\right)}-1\;vs.\;ln{\dot{\varepsilon }}^{*}$ and (**b**) $ln\left(1-\frac{\sigma }{\left(A_{j}+B_{j}\varepsilon^{n_{j}}\right)}\right)\;vs.\;ln{T_{j}}^{*}$ at strain range (0.2–0.6) to evaluate $C_{j}$ and $m_{j}$, respectively.

After that, at the reference temperature, $C_{j}$ could be estimated using Equation (22) by taking the mean slope value of the linear fit of the curve $\frac{\sigma }{\left(A_{j}+B\varepsilon^{n_{j}}\right)}-1\;vs.\;ln{\dot{\varepsilon }}^{*}$, as shown in Figure 9a.

Equation (20) can be rewritten as follows by multiplying both sides by the natural logarithm at the reference strain rate:

(23) $$ln\left(1-\frac{\sigma }{\left(A_{j}+B_{j}\varepsilon^{n_{j}}\right)}\right)=m_{j}ln{T_{j}}^{*}$$

In the next step, at the reference strain rate, $m_{j}$ was estimated using Equation (23) by taking the mean slope value of the linear fit of the curve $ln\left(1-\frac{\sigma }{\left(A_{j}+B_{j}\varepsilon^{n_{j}}\right)}\right)\;vs.\;ln{T_{j}}^{*}$, as shown in Figure 9b. Since there are not available data at 1173 K for this curve, it was assumed that the reference temperature would be changed from 1173 K to 1123 K.

Using Figure 8 and Figure 9, the values of $A_{j}$ (MPa), $n_{j}$, $B_{j}$, $C_{j}$, and $m_{j}$ at the strain range (0.2–0.6) could be evaluated as listed in Table 3. By substituting all the values of the constants, $A_{j}$, $n_{j}$, $B_{j}$, $C_{j}$, corresponding strain and strain rate values, and ${T_{j}}^{*}$ into Equation (20), the anticipated flow stress values for all strain rates, temperatures, and strain ranges may be calculated.

**Figure 10:**
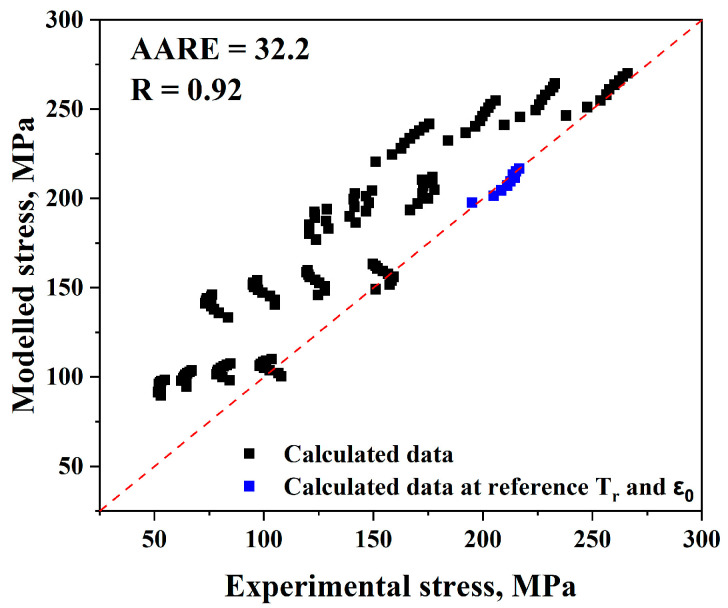
Comparison of the Johnson–Cook model’s anticipated and experimental stress data.

Therefore, it is possible to verify the validity of the Johnson–Cook model by comparing the experimental and anticipated flow stress levels using Equations (18) and (19), as shown in Figure 10. For the Johnson–Cook model, (*AARE*) and (*R*) are equal to 32.2% and 0.92, respectively.

**Figure 11:**
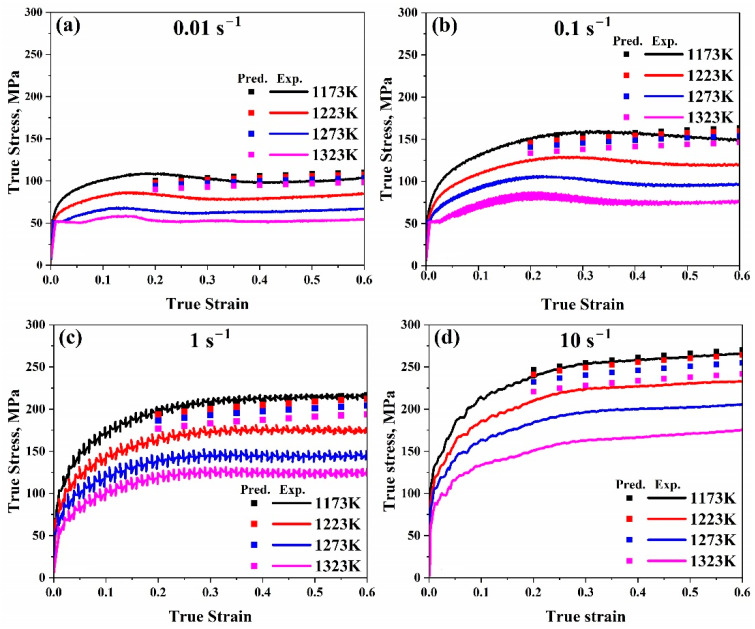
Comparison of the Johnson–Cook model’s true strain–true stress anticipated and experimental flow stress data at temperature range (1173–1323 K) at (**a**) 0.01 s^−1^, (**b**) 0.1 s^−1^, (**c**) 1 s^−1^, and (**d**) 10 s^−1^, respectively.

As illustrated in Figure 11, the strain–stress experimental curves could be compared with the predicted flow stress values after evaluation.

### 3.3. Modified Johnson–Cook Model (Modified J–C)

The following formula can be used to depict the modified version of the Johnson–Cook model [17]:

(24) $$\sigma =\left(P_{j}+Q_{j}\varepsilon^{n_{j}^{\prime }}\right){{\dot{\varepsilon }}^{*}}^{r}\left[1+\left(\frac{\sigma_{m}}{\sigma_{y}}-1\right)exp\left(-\alpha_{j}{{T_{j}}^{*\prime }}^{\beta_{j}}\right)\right]$$

where ε is the strain; σ is the flow stress (MPa); ${\dot{\varepsilon }}^{*}$ is the dimensionless strain rate $\left({\dot{\varepsilon }}^{*}=\dot{\varepsilon }/{\dot{\varepsilon }}_{ο}\right)$; $\dot{\varepsilon }$ is the strain rate (s^−1^); ${\dot{\varepsilon }}_{ο}$ is the reference strain rate (s^−1^); ${T_{j}}^{*\prime }$ equals to 0$\left(T_{m}-T\right)/\left(T-T_{r}\right)$; T is the current temperature (K); $T_{m}$ is the melting temperature (K); $T_{r}$ is the reference temperature (K); $P_{j}$ is the yield strength at the reference strain rate and temperature; and $Q_{j},n_{j}^{\prime },r,\;\alpha_{j},\beta_{j}$ are material constants. In order to solve this model, similarly to the original one, it was necessary to identify the reference strain rate, temperature, yield stress at these points, and melting point for AISI 8822H steel. These values were found to be 1 s^−1^, 1173 K, 60 MPa, and 1743 K, respectively.

Equation (24) can be rewritten as follows by multiplying both sides by the natural logarithm at the reference conditions:

(25) $$\ln\;\left(\sigma -P_{j}\right)=lnQ_{j}+n_{j}^{\prime }ln\varepsilon$$

**Figure 12:**
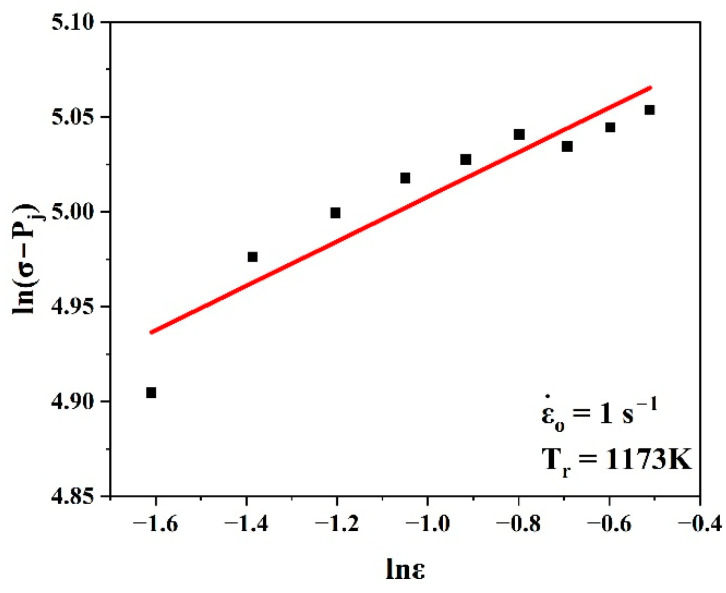
Plots of $ln(\sigma -P_{j})\;vs.\;ln\varepsilon$ to evaluate $n_{j}^{\prime }$ and $lnQ_{j}$.

Equation (25) was used to evaluate $n_{j}^{\prime }$ and $lnQ_{j}$ by taking the slope and intercept of the linear fit of the curve $ln(\sigma -P_{j})\;vs.\;ln\varepsilon$, as shown in Figure 12. Hence, it was simple to evaluate $Q_{j}$.

Equation (24) can be modified to the following at reference temperature:

(26) $$ln\left(\frac{\sigma }{P_{j}+Q_{j}\varepsilon^{n_{j}^{\prime }}}\right)=rln{\dot{\varepsilon }}^{*}$$

**Figure 13:**
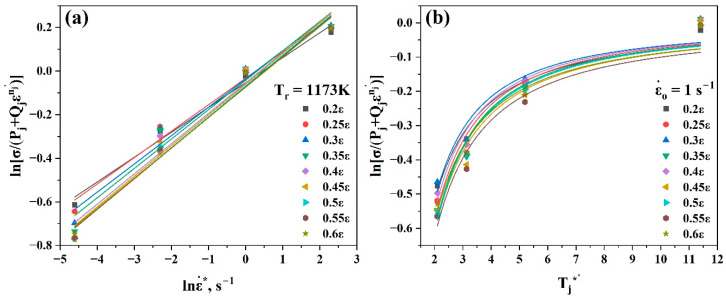
Plots of (**a**) $ln\left(\frac{\sigma }{P_{j}+Q_{j}\varepsilon^{n_{j}^{\prime }}}\right)\;vs.\;ln{\dot{\varepsilon }}^{*}$ and (**b**) $ln\left(\frac{\sigma }{P_{j}+Q_{j}\varepsilon^{n_{j}^{\prime }}}\right)\;vs.\;{T_{j}}^{*\prime }$ at strain range (0.2–0.6) to evaluate r, $\alpha_{j}$, and $\beta_{j}$, respectively.

After that, at the reference temperature, r could be estimated using Equation (26) by taking the mean slope value of the linear fit of the curve $ln\left(\frac{\sigma }{P_{j}+Q_{j}\varepsilon^{n_{j}^{\prime }}}\right)\;vs.\;ln{\dot{\varepsilon }}^{*}$, as shown in Figure 13a.

Equation (24) can be rewritten as follows by multiplying both sides by the natural logarithm at the reference strain rate:

(27) $$ln\left(\frac{\sigma }{P_{j}+Q_{j}\varepsilon^{n_{j}^{\prime }}\left({{\dot{\varepsilon }}^{*}}^{r}\right)}\right)=-\alpha_{j}{{T_{j}}^{*\prime }}^{\beta_{j}}$$

In the next step, at the reference strain rate, $\alpha_{j}$ and β were estimated using Equation (27) by taking the first-order power function fit of the curve $ln\left(\frac{\sigma }{P_{j}+Q_{j}\varepsilon^{n_{j}^{\prime }}}\right)\;vs.\;{T_{j}}^{*\prime }$, as shown in Figure 13b.

Similarly to the Johnson–Cook model, the reference temperature was changed from 1173 K to 1123 K. Using Figure 8 and Figure 9, the values of $P_{j}$ (MPa), $n_{j}^{\prime }$, $Q_{j}$, r, $\alpha_{j}$, and $\beta_{j}$ at the strain range (0.2–0.6) could be evaluated as listed in Table 4. By substituting all the values of the constants, $P_{j}$, $n_{j}^{\prime }$, $Q_{j}$, r, $\alpha_{j}$, $\beta_{j}$, corresponding strain and strain rate values, and ${T_{j}}^{*}$ into Equation (24), the anticipated flow stress values for all strain rates, temperatures, and strain ranges may be calculated.

**Figure 14:**
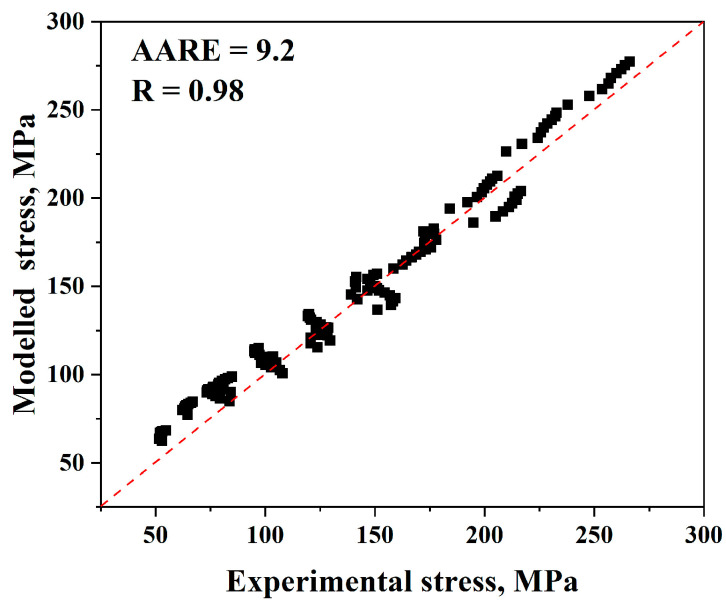
Comparison of the modified Johnson–Cook model’s anticipated and experimental stress data.

It is therefore possible to verify the validity of the Johnson–Cook model by comparing the experimental and anticipated flow stress levels using Equations (18) and (19), as shown in Figure 14. For the modified Johnson–Cook model, (*AARE*) and (*R*) are equal to 9.2% and 0.98, respectively.

**Figure 15:**
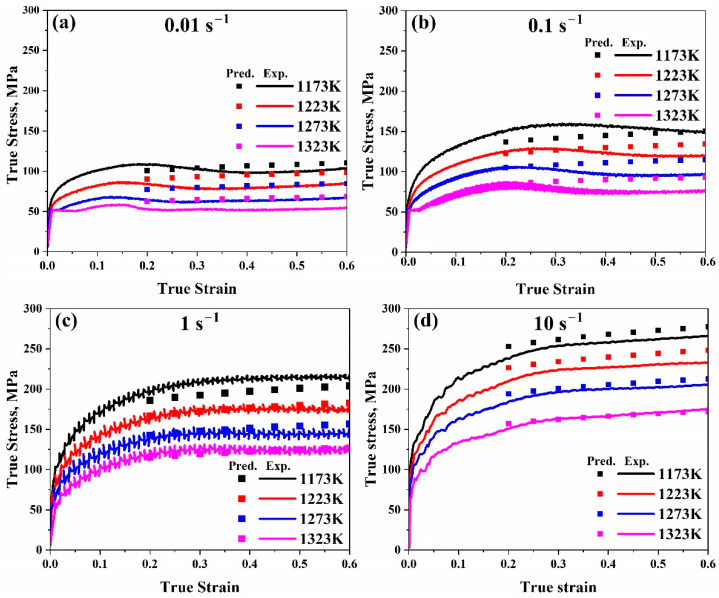
Comparison of the modified Johnson–Cook model’s true strain–true stress anticipated and experimental flow stress data at temperature range (1173–1323 K) at (**a**) 0.01 s^−1^, (**b**) 0.1 s^−1^, (**c**) 1 s^−1^, and (**d**) 10 s^−1^, respectively.

As illustrated in Figure 15, the true strain–true stress experimental curves could be compared with the predicted flow stress values after evaluation.

### 3.4. Trimble Model

The Trimble model is defined by the following formula [18]:

(28) $$\sigma =A_{t}\varepsilon^{n_{t}}exp\left(B_{t}\varepsilon +C_{t}\right){T_{t}}^{*}$$

where σ is the flow stress (MPa); ε is the strain; ${T_{t}}^{*}$ equals to $\left(T-T_{r}\right)$; T is the current temperature (K); $T_{r}$ is the reference temperature (K); and $A_{t},n_{t},B_{t},\;C_{t}$ are material constants. In contrast to the Johnson–Cook models, this model started with a reference temperature of 1123 K.

Equation (24) can be rewritten as follows by multiplying both sides by the natural logarithm:

(29) $$ln\sigma =lnA_{t}+n_{t}ln\varepsilon +\left(B_{t}\varepsilon +C_{t}\right){T_{t}}^{*}$$

For each strain value at one particular strain rate, to be able to solve this model, two additional parameters were assumed, as mentioned in Equations (30) and (31):

(30) $$S_{t}=B_{t}\varepsilon +C_{t}$$

(31) $$I_{t}=lnA_{t}+n_{t}ln\varepsilon$$

**Figure 16:**
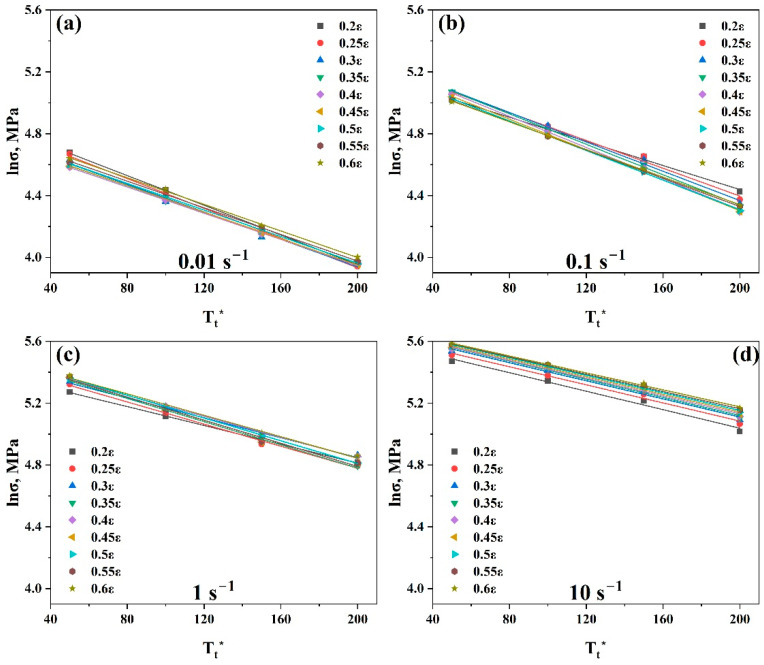
Plots of $\left(ln\sigma \;vs.\;{T_{t}}^{*}\right)$ at strain range (0.2–0.6) at (**a**) 0.01 s^−1^, (**b**) 0.1 s^−1^, (**c**) 1 s^−1^, and (**d**) 10 s^−1^ to evaluate $S_{t}$ and $I_{t}$, respectively.

Equation (29) was used to evaluate $S_{t}$ and $I_{t}$ by taking the slope and intercept of the linear fit of the curve $ln\sigma \;vs.\;{T_{t}}^{*}$ at the strain range (0.2–0.6), as shown in Figure 16.

**Figure 17:**
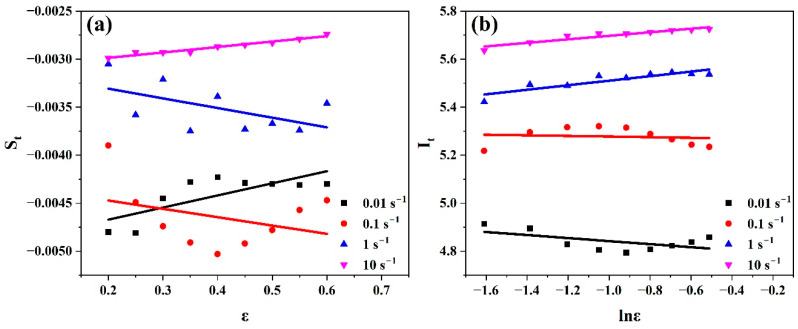
Plots of (**a**) $\left(S_{t}\;vs.\;\varepsilon \right)$ to evaluate $B_{t}$ and $C_{t}$ and (**b**) $\left(I_{t}\;vs.\;ln\varepsilon \right)$ to evaluate $n_{t}$ and $lnA_{t}$ at strain rate range (0.01–10 s^−1^).

Equation (30) was used to evaluate $B_{t}$ and $C_{t}$ by taking the slope and intercept of the linear fit of the curve $\left(S_{t}\;vs.\;\varepsilon \right)$, as shown in Figure 17a.

Equation (30) was used to evaluate $n_{t}$ and $lnA_{t}$ by taking the slope and intercept of the linear fit of the curve $I_{t}\;vs.\;ln\varepsilon$, as shown in Figure 17b. Hence, it is simple to evaluate $A_{t}$.

Using Figure 17, the values of $B_{t}$, $C_{t}$, $n_{t}$, and $A_{t}$ at the strain rate range (0.01–10 s^−1^) could be evaluated as listed in Table 5.

By substituting all the values of the constants, $B_{t}$, $C_{t}$, $n_{t}$, $A_{t}$ corresponding strain values, and ${T_{t}}^{*}$ into Equation (29), the anticipated flow stress values for all strain rates, temperatures, and strain ranges may be calculated.

**Figure 18:**
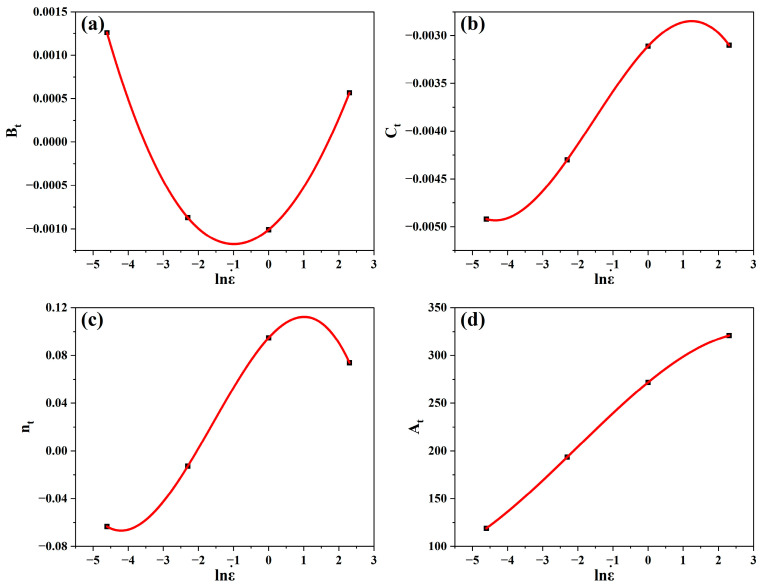
Polynomial fitting of (**a**) $B_{t}\;vs.\;ln\dot{\varepsilon }$, (**b**) $C_{t}\;vs.\;ln\dot{\varepsilon }$, (**c**) $n_{t}\;vs.\;ln\dot{\varepsilon }$, and (**d**) $A_{t}\;vs.\;ln\dot{\varepsilon }$.

A polynomial fit was produced for each constant evaluated under different strain rates, and it was demonstrated that the best match was achieved when the third order of polynomials was used, as illustrated in Figure 18.

As a result, the regression in the equations of the relevant material constants $B_{t}$, $C_{t}$, $n_{t}$, and $A_{t}$ as a function of strain rate was reasonably described by Equations (32)–(35), respectively.

(32) $$B_{t}=-0.001+{3.32*10}^{-4}ln\dot{\varepsilon }+{1.62*10}^{-4}ln{\dot{\varepsilon }}^{2}-{3.73*10}^{-6}ln{\dot{\varepsilon }}^{3}$$

(33) $$C_{t}=-0.0031+{3.87*10}^{-4}ln\dot{\varepsilon }-{1.11*10}^{-4}ln{\dot{\varepsilon }}^{2}-{2.39*10}^{-5}ln{\dot{\varepsilon }}^{3}$$

(34) $$n_{t}=0.0947+0.0322ln\dot{\varepsilon }-0.0121ln{\dot{\varepsilon }}^{2}-0.0025ln{\dot{\varepsilon }}^{3}$$

(35) $$A_{t}=271.8+30.07ln\dot{\varepsilon }-2.76ln{\dot{\varepsilon }}^{2}-0.4516ln{\dot{\varepsilon }}^{3}$$

**Figure 19:**
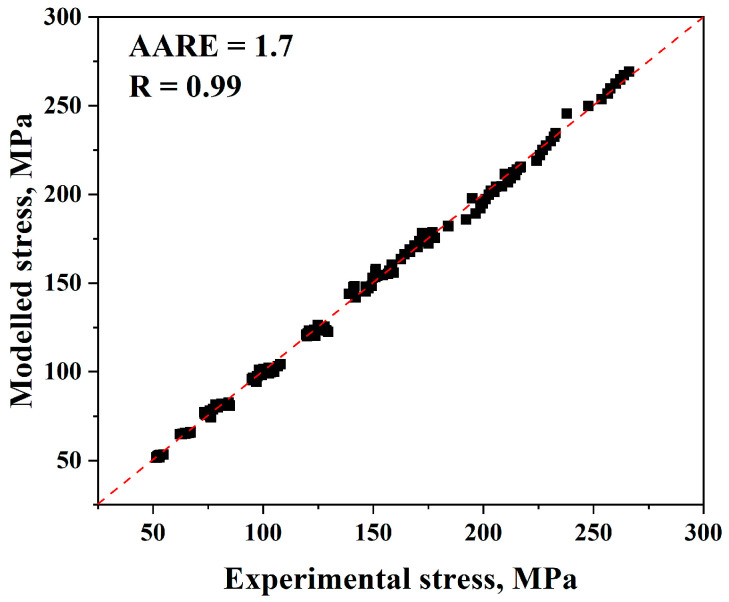
Comparison of the Trimble model’s anticipated and experimental stress data.

It is therefore possible to verify the validity of the Trimble model by comparing the experimental and anticipated flow stress levels using Equations (18) and (19), as shown in Figure 19. For the Trimble model, (*AARE*) and (*R*) are equal to 1.7% and 0.99, respectively.

**Figure 20:**
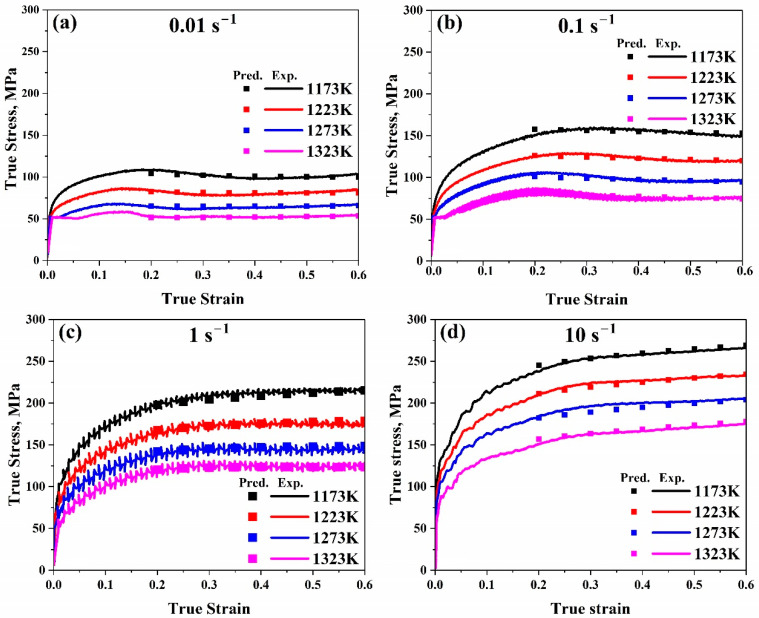
Comparison of the Trimble model’s true strain–true stress anticipated and experimental stress data at temperature range (1173–1323 K) at (**a**) 0.01 s^−1^, (**b**) 0.1 s^−1^, (**c**) 1 s^−1^, and (**d**) 10 s^−1^, respectively.

As illustrated in Figure 20, the true strain–true stress experimental curves could be compared with the predicted flow stress values after evaluation.

## 4. Discussion

The Arrhenius-type model is one of the most widely used constitutive models for predicting flow stress values in hot deformation testing. After comparing our experimental and anticipated data, it was observed from Figure 6 that the results are very close, which confirms that this model is quite accurate at elevated temperatures. Furthermore, by contrasting the measured curves for a true strain–true stress with the anticipated flow stress values, as illustrated in Figure 7, it is observed that for all ranges, predicted data are very close to the experimental ones, proving that this model is accurate to predict our data. Furthermore, it can be found that (*AARE*) is too low (2.6%) and (*R*) is too high (0.99). Taking into account that the lower the (*AARE*) and the higher the *R*, the more accurate the model, it can be determined that, in general, among the models that forecast flow stress values, one of the most accurate is the Arrhenius-type.

For the Johnson–Cook model, it is evident from Figure 10 that experimental and predicted data only exhibit good consistency when the strain rate and reference temperature are met; under all other circumstances, there are significant deviations. Moreover, by making a comparison between true strain–true stress experimental and predicted flow stress values, it was observed from Figure 11 that there is a significant variation in the data as the temperature rises, even though the general pattern in the experimental data curve and the predicted value match. Additionally, the more the temperature, strain rate, and reference value vary, the more significant the variation between the experimental and anticipated data.

The main reason for this is that an accurate and successful model of metals should take into consideration the impacts of the three distinct forms of softening, in contrast to the Johnson–Cook constitutive model, which treats them as different elements [16]. Researchers who have examined this topic have discovered that these three impacts are coupled. The coupling effect, which can result in nonlinear changes in the rheological behaviour of metals depending on the situation, is caused by the intricate interaction between dislocation build-up and recovery mechanisms throughout plastic deformation.

Specifically, the temperature softening effect and transient strain rate may interact positively or negatively, while the material’s various microstructural properties and processing conditions cause the strain hardening effect. Thus, compounding the impact of several components cannot adequately capture the complicated plastic deformation behaviour. However, some metals are more vulnerable to the coupling impact of many factors. The study results show that this coupling impact is evident in the plastic deformation of AISI 8822H steel at high temperatures. Therefore, to construct a constitutive model of AISI 8822H steel with more accuracy throughout the plastic deformation, the above model must be altered.

Furthermore, it can be found that (*AARE*) is too high (32.2%) and (*R*) is too low (0.92). Therefore, it may be concluded that the Johnson–Cook model is generally not a reliable model for predicting flow stress values.

**Figure 21:**
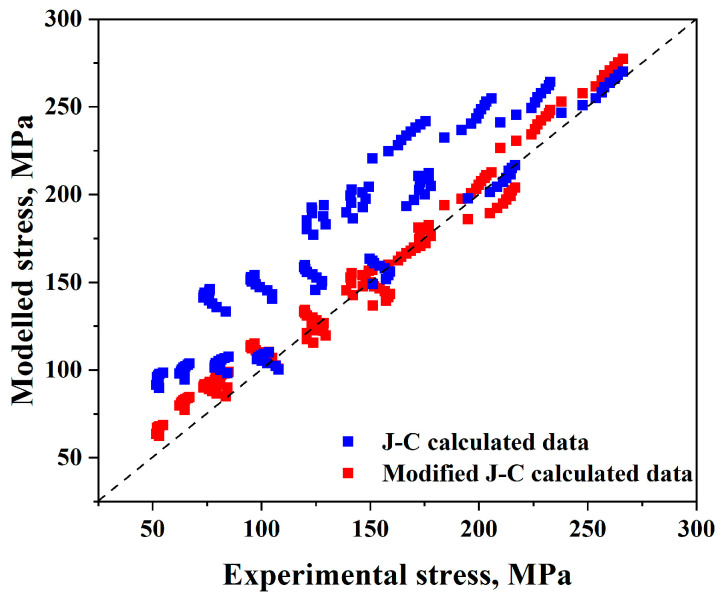
Comparison of the experimental and anticipated stress data for the original and modified Johnson–Cook models.

More recent constitutive models have focused on developing or modifying new models. According to experimental hot compression values, a modified model explains the flow stress behaviour of the AISI 8822H alloy. In most cases, the updated model was found to have a higher correlation with experimental data than the original model, as illustrated in Figure 21.

As illustrated in Figure 14, the updated model’s anticipated flow stress values are excessively near the experimental values, increasing the model’s accuracy.

Furthermore, as illustrated in Figure 15, contrasting the measured curves for a true strain–true stress with the anticipated flow stress values reveals that for all ranges, the predicted data for the modified model are significantly closer to the experimental ones than the original one. This demonstrates how well the updated model predicts our data compared to the original.

Additionally, it is discovered that the (*AARE*) and (*R*) values are equal to 9.2% and 0.98, respectively. Hence, it can be determined that, in general, the modified Johnson–Cook model is clearly more accurate than the original one for predicting flow stress values.

The Trimble model is the newest model used to forecast flow stress data. After comparing our experimental and anticipated data in the Trimble model, it was observed from Figure 19 that the results are very close, which confirms how accurate it is. Furthermore, by contrasting the measured curves for a true strain–true stress with the anticipated flow stress values, as illustrated in Figure 20, it is observed that for all ranges, predicted data are very close to the experimental ones, which again proves that this model is accurate for predicting our data. Furthermore, it can be found that (*AARE*) is too low (1.7%) and (*R*) is too high (0.99). Therefore, the Trimble model is one of the best models for predicting flow stress levels.

After evaluating all constitutive equations and flow stress values of the four models, it is now time to compare each one to identify the most accurate and appropriate model for assessing the anticipated flow stress values.

**Figure 22:**
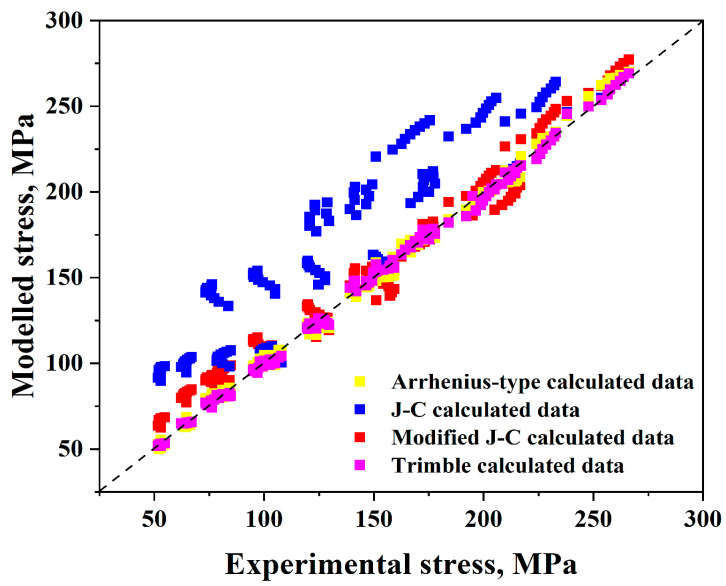
Comparison of the experimental and anticipated stress data for the four models.

First, the experimental and expected flow stress values for each of these models were compared, as illustrated in Figure 22. This figure shows that the Arrhenius-type and Trimble models are the most constitutive accurate ones, while the Johnson–Cook model is the least accurate one for forecasting the flow stress values.

Additionally, the modified Johnson–Cook model is much better than the original but less accurate than the Arrhenius-type and Trimble models.

**Figure 23:**
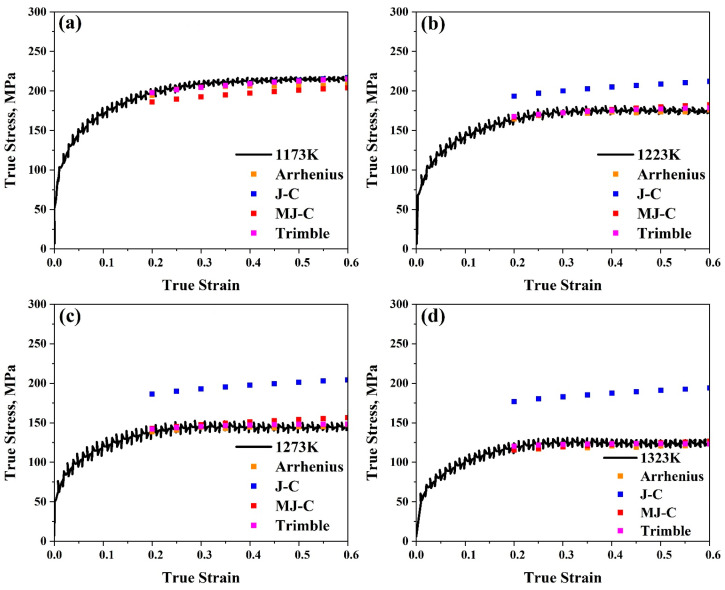
Comparison between true strain–true stress experimental curves and predicted flow stress values for the Arrhenius-type, Johnson–Cook, modified Johnson–Cook, and Trimble models at 1 s^−1^ at (**a**) 1173 K, (**b**) 1223 K, (**c**) 1273 K, and (**d**) 1323 K, respectively.

**Figure 24:**
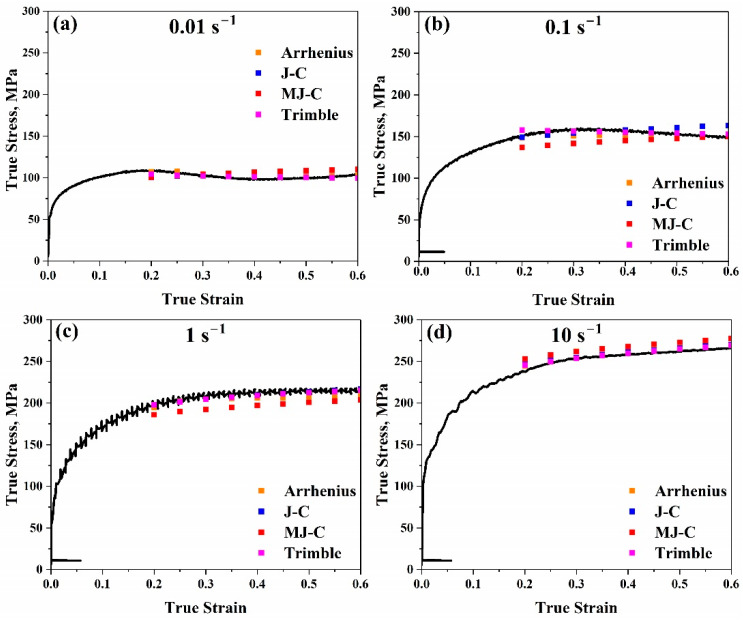
Comparison between true strain–true stress experimental curves and modelled flow stress values for the Arrhenius-type, Johnson–Cook, modified Johnson–Cook, and Trimble models at 1173 K at (**a**) 0.01 s^−1^, (**b**) 0.1 s^−1^, (**c**) 1 s^−1^, and (**d**) 10 s^−1^, respectively.

Furthermore, by making a comparison between true strain–true stress experimental data and predicted flow stress values for these models, in our study, the behaviour of flow stress of AISI 8822H steel was checked concerning two cases. The first case was applied at the range (1173–1323 K) with a particular strain rate value of 1 s^−1^, for example, as illustrated in Figure 23. The second case was applied at the range (0.01–10 s^−1^) with a specific temperature value of 1173 K, for example, as shown in Figure 24. The Arrhenius-type and Trimble models are the most accurate models since the variation between the anticipated and experimental values in both models is always too low for all ranges. The Johnson–Cook model is accurate at all strain rate values, especially at 1173 K, since the variation between the anticipated and experimental data is low. The higher the temperature, the lower the accuracy since the variation between the anticipated and experimental data becomes higher and higher for all strain rate values. The weak accuracy of the Johnson–Cook model is improved by using the modified model, particularly at temperatures over 1173 K. Consequently, it is evident that the variation between the anticipated and experimental data in this model is always too low for all strain rates and temperature values higher than 1173 K.

One more approach to comparing the four models is to compare the values of (*AARE*) and (*R*), as represented in Table 6. This table clearly observes that the smallest value of (*AARE*) and the largest value of (*R*) exist in the Trimble model, which emphasises that, for AISI 8822H steel, the most accurate model to assess the anticipated flow stress values is the Trimble model.

## 5. Conclusions

The following observations were developed after consideration of the compression tests performed on axisymmetric samples and an investigation of the test data that were obtained:

(1) The flow stress values for AISI 8822H steel increased when the strain rate increased at a constant temperature and when the deformation temperature decreased at a constant strain rate.
(2) Based on the results of (*AARE*) and (*R*) for the four constitutive models, the Trimble model was found to have the highest (*R*) value, which equals 0.99, and the lowest *AARE* value, which equals 1.7%. Consequently, the Trimble model is the most suitable for predicting the hot deformation behaviour of AISI 8822H steel over the processing range investigated in this study.
(3) The Johnson–Cook model was found to have the lowest (*R*) value, 0.92, and the highest *AARE* value, 32.2%. Consequently, the Johnson–Cook model is the least suitable for predicting the hot deformation behaviour of AISI 8822H steel over the processing range investigated in this study.

## Figures and Tables

**Table 1:** AISI 8822H steel’s chemical composition in weight percentage.

C	Mn	Si	Cr	Ni	Mo	Cu	Co	Fe
0.27	0.94	0.25	0.44	0.63	0.36	0.17	0.01	Bal.

**Table 2:** Values of the constants at 0.4 ε for the Arrhenius-type model.

$n^{\prime }$	β	α	n	$Q_{def}$	A
6.2	0.05	0.008	4.6	316.5	2 × 10^12^

**Table 3:** Values of the constants at strain range (0.2–0.6) for the Johnson–Cook model.

$A_{j}$	$n_{j}$	$B_{j}$	$C_{j}$	$m_{j}$
60	0.117	168.188	0.107	1.935

**Table 4:** Values of the constants at strain range (0.2–0.6) for the modified Johnson–Cook model.

$P_{j}$	$n_{j}^{\prime }$	$Q_{j}$	r	$\alpha_{j}$	$\beta_{j}$
60	0.12	168.19	0.13	1.36	−1.23

**Table 5:** Values of the constants at strain rate range (0.01–10 s^−1^) for the Trimble model.

$\dot{\varepsilon }$ (s^−1^)	Constants			
	$B_{t}$	$C_{t}$	$n_{t}$	$A_{t}$
0.01	0.0013	−0.0049	−0.0633	118.96
0.1	−0.0009	−0.0043	−0.0127	193.46
1	−0.001	−0.00311	0.0947	271.8
10	−0.0006	−0.0031	0.0739	320.91

**Table 6:** Comparison between the Arrhenius-type, Johnson–Cook, modified Johnson–Cook, and Trimble models with respect to *ARRE* % and *R*.

	Constitutive Models			
	Arrhenius-Type	Johnson–Cook	Modified Johnson–Cook	Trimble
*ARRE* %	2.6	32.2	9.2	1.7
*R*	0.99	0.92	0.98	0.99

## Data Availability

The generated data can be obtained from wojciech.borek@polsl.pl or ke900594@student.polsl.pl.
